# Structure and Relationships of University Instructors’ Achievement Goals

**DOI:** 10.3389/fpsyg.2016.00375

**Published:** 2016-03-23

**Authors:** Martin Daumiller, Robert Grassinger, Oliver Dickhäuser, Markus Dresel

**Affiliations:** ^1^Department of Psychology, University of AugsburgAugsburg, Germany; ^2^Department of Educational Psychology, University of MannheimMannheim, Germany

**Keywords:** achievement goals, goal orientation, university, teaching, motivation

## Abstract

The present study examines the achievement goals of university instructors, particularly the structure of such goals, and their relationship to biographic characteristics, other aspects of instructors’ motivation, and teaching quality. Two hundred and fifty-one university instructors (184 without Ph.D., 97 with Ph.D., thereof 51 full professors; 146 males, 92 females) answered a questionnaire measuring achievement goals, self-efficacy, and enthusiasm in altogether 392 courses. Teaching quality was assessed using reports from 9,241 students who were attending these courses. Confirmatory factor analyses revealed mastery, performance approach, performance avoidance, work avoidance, and relational goals as being distinguishable from each other. Distinct relationships were found between different instructors’ achievement goals, and gender, age, and career status as well as self-efficacy and enthusiasm. Hierarchical linear models suggested positive associations of instructors’ mastery goals with teaching quality, while negative associations were indicated for performance avoidance goals and work avoidance goals in relation to teaching quality. Exploratory analyses conducted due to a quite large correlation between performance approach and performance avoidance goals indicated that for university instructors, differentiating performance goals into appearance and normative components might also be adequate. All in all, the study highlights the auspiciousness of the theoretical concept of university instructors’ achievement goals and contributes to making it comprehensively accessible.

## Introduction

High quality of teaching is regarded as a central premise for students’ development of competences in tertiary education (Norton et al., 2005). It can be assumed that the instructional methods instructors adopt in higher education play a key role in student attainment and, ultimately, in the success of the whole institution (Biggs, 1999). Hereby, instructors’ motivation constitutes a fundamental prerequisite for developing teaching quality (Rindermann, 2009). However, few studies have investigated the motivational forces of university instructors. They underpin the significance of motivation for university teaching by indicating that strong motivation is associated with good teaching quality and teaching results as well as the use of teaching development opportunities (Young and Kline, 1996; Morris and Usher, 2011). However, these studies do not include socio-cognitive aspects of motivation and, most importantly, regard motivation as a unidimensional construct; they follow the question of “how much” university instructors are motivated. From current educational psychological perspectives, the question of the quality of motivation is of primary relevance (Pintrich, 2000b).

In light of this understanding, achievement goal theory has been developed, and entrenched itself as a central and prolific concept of educational psychological motivation research in the last decades (de la Fuente, 2004; Elliot, 2005; Maehr and Zusho, 2009; Hulleman et al., 2010). *Achievement goals* can be defined as cognitive representations of results or end states in achievement-related tasks and settings that the individual is committed to either approach or avoid (Austin and Vancouver, 1996; Maehr and Zusho, 2009; Hulleman et al., 2010). Research clearly demonstrated that different achievement goals have different cognitive, affective, and behavioral consequences (Payne et al., 2007; Hulleman et al., 2010).

Recently, much evidence has been provided showing that achievement goals are also important for the experiences and behaviors of school teachers at work (Butler, 2007; Nitsche et al., 2011; Retelsdorf and Günther, 2011). In agreement, it can be expected that addressing university instructors’ achievement goals is useful for understanding the motivations underlying instructional practices (Daumiller et al., 2015). However, research on the achievement goals of school teachers is not transferable to the context of higher education without further consideration. The present research investigates the achievement goals of university instructors in a quantitative study, and examines the structure and relationships of such goals with other aspects of instructors’ teaching motivation and teaching quality as perceived by their students.

## University Instructor Achievement Goals

In educational settings, achievement goal theory has primarily been studied with respect to student populations (Senko et al., 2011). More recently, however, this concept has been expanded to school teachers (Butler, 2007; Fasching et al., 2011; Retelsdorf and Günther, 2011; Dresel et al., 2013). Similarly to schools, universities are also regarded as contexts that require constant learning and improvement (achievement arenas for both pupils and teachers; cf. Butler, 2007, p. 242). In this light, university instructors are expected to face challenges similar to learners in any context. As such, achievement goal theory can be regarded as a potentially fruitful concept to gain further insight into the quality of instructors’ motivation (Maehr and Zusho, 2009). Research on the achievement goals of school teachers—which addresses achievement goals’ factorial structures and relationships with teacher (e.g., acquisition of competence, experience of stress and work-load) and student variables (e.g., learning gains)—constitutes the starting point of the present work.

While research on teachers in primary and secondary education has more frequently covered the concept of achievement goals, there is hardly any research addressing the achievement goals of university instructors. The only few existing studies show that employees at higher education facilities pursue distinct personal goals that can be classified in distinct goal profile types based on their focus: teaching, students, or self (Wosnitza et al., 2014). However, there are first indications that the concept of achievement goals is suitable to describe university instructors’ motivation: In a qualitative interview study by Daumiller et al. (2015), university instructors considered achievement goals central to their teaching related aims and reported pursuing multiple goals.

Systemic similarities and differences between primary and secondary schools, and universities imply that research findings on school teachers need to be verified and differentiated for the university context. Since both school teachers and university instructors have an official teaching assignment and arrange teaching/learning settings for their students, it can be assumed that respective insights from school-based research principally also apply to university instructors. However, university instructors usually have, apart from their teaching activities, additional time-intensive tasks (e.g., research, administration), and more freedom in the organization and content of their courses than school teachers. Furthermore, there are also structural differences in regard to their addressees—university students usually have more experience, higher motivation, and stronger interest than school students (Marsh, 2007). Due to these systemic differences in the content and structure of demands and opportunities, it can be assumed that university instructors may pursue different achievement goals than school teachers and that these can be reached by different means and have different consequences.

### Structure and Classes of Goals

In order to describe the structure of achievement goals in the teaching profession, a model has been developed that differentiates between five superordinate classes of goals (Butler, 2012). *Mastery goals* are goals that are directed at the acquisition and development of own competences (e.g., improving one’s teaching skills). *Performance approach goals* place focus on the demonstration of own competences (e.g., appearing as competent in front of colleagues or students). *Performance avoidance goals* are directed at the avoidance of failure and the demonstration of inferior competences (e.g., concealing a lack of knowledge). *Work avoidance goals* reflect strivings to get through the day with little effort, and *relational goals* describe the aspiration of creating close and caring relationships with students.^1^ These five goal classes have been shown to be associated with school teachers’ acquisition of competence and instructional practices (Butler and Shibaz, 2008; Retelsdorf et al., 2010; Dresel et al., 2013), attitudes toward further training and professional learning (Butler, 2007; Runhaar et al., 2010; Nitsche et al., 2013b), and experience of stress and workload (Tönjes and Dickhäuser, 2009).

Although there is general consensus about the existence and structure of these achievement goal classes in the literature, current meta-analyses reach the conclusion that often the same label is used for conceptually different constructs (Hulleman et al., 2010). This is especially the case for performance goals. In various reviews of literature, two basic defining components have been identified which might be differently related to performance outcomes (Elliot, 1999, 2005; Urdan and Mestas, 2006), namely an appearance component (demonstration and affirmation of competences to an audience, e.g., wanting others to think one is competent), and a normative component (an explicit normative comparison, e.g., wanting to be more competent than others). Since conceptual clarity and, subsequently, measurement (i.e., operational) consistency are essential (Shadish, 2002), it is important to carefully delineate the theoretical underpinnings of these goals when examining and measuring them, i.e., items should consciously be chosen or balanced concerning the above mentioned components.

Daumiller et al. (2015) addressed the achievement goals of university instructors using qualitative interviews. Results indicated that mastery, performance approach, performance avoidance, work avoidance, and relational goals were reported by university instructors, and that substantial individual differences exist in the importance of all goal classes. For performance goals, Daumiller et al. (2015) found goals on both the appearance and the normative level—e.g., when asked about their personal goals, some instructors reported that it was important for them to be perceived as very good instructors (appearance component), whereas other instructors mentioned their striving to be better than other instructors (normative component). The presence of appearance and normative aspects for both performance approach and performance avoidance goals indicates that this differentiation might be important for the university context.

Based upon these findings, it can be expected that university instructors’ achievement goals are relevant and comprise at least mastery, performance approach, performance avoidance, relational, and work avoidance goals. For the present study, we primarily pursue the well-established distinction of performance goals in their approach and avoidance component (however, we acknowledge that appearance and normative aspects may not be omitted).

### Achievement Goals, and Age, Gender, and Career Status

Different achievement goals are regarded as facilitative for different groups of people (Elliot, 1999). Research also shows that achievement goals vary depending on individual factors such as age, gender, and competence level (Harackiewicz et al., 1998; Midgley et al., 2001; Nitsche et al., 2013a). Therefore, we also expect different achievement goals of university instructors to be of varying importance depending on their age, gender, and career status. In line with the results on gender effects for achievement goals for students and school teachers (e.g., Kenney-Benson et al., 2006; Nitsche et al., 2013a), we generally expect women to have stronger mastery goals and less performance approach, performance avoidance and work avoidance goals than men. It seems reasonable to assume that these differences also prevail for university instructors: For example, since female university instructors often feel more pressure to further develop their competencies (Acker and Feuerverger, 1996; Acker and Armenti, 2004), they might also develop stronger mastery goals. Following the theoretical rationale of Butler (2014) that women endorse intimacy and improvement of social development goals to a greater extent than men, we also expect higher relational goals for women. In addition to these gender differences, we expect mastery goals to be less important for full professors (since they have finished their qualification already), and work avoidance goals to be pursued to a larger extent by younger instructors (since they often have more and frequently conflicting tasks: For example, younger instructors often regard teaching as a burden next to research, whilst it is found that when approaching retirement, there is an increased interest in teaching amongst university instructors; Baldwin and Blackburn, 1981).

### Achievement Goals and Other Aspects of Instructors’ Teaching Motivation

Researchers have consistently assumed and found that while especially performance avoidance goals are associated with maladaptive patterns of learning (e.g., surface learning, low interest) and low achievement, mastery goals are associated with adaptive patterns of learning (e.g., use of deep-processing learning strategies, high interest) and high achievement (Anderman and Young, 1994; Midgley and Urdan, 1995; Middleton and Midgley, 1997; Pajares et al., 2000). Achievement goals are also related differently to other aspects of motivation, such as self-efficacy and enthusiasm—which are frequently used to characterize the cognition and behavior of teachers and instructors (Stajkovic and Luthans, 1998; Kunter et al., 2011). Thus, they form important variables within the nomological network of motivational constructs to be investigated in the present study.

Teachers’ self-efficacy, defined as a teacher’s judgment of his or her capabilities to organize and successfully complete tasks in instructional contexts (Bandura, 1997), has been shown to be associated with various motivational and behavioral processes, such as being more resilient when facing obstacles, planning, and organizing courses more effectively, and employing more engaging instructional strategies (Tschannen-Moran et al., 1998; Gencer and Cakiroglu, 2007). Therefore, it can be assumed that self-efficacy beliefs are also associated with the teaching quality of university instructors (cf. Morris and Usher, 2011). The relation with achievement goals has mainly been addressed for student populations. Thereby, mastery goals have consistently and positively been associated with self-efficacy (Ames, 1992; Pintrich, 2000a), performance avoidance goals have been negatively associated with self-efficacy (Middleton and Midgley, 1997), and performance approach goals have been inconsistent in regard to associations (i.e., some studies reporting positive associations with self-efficacy and some studies reporting negative associations).

Enthusiasm is a mental state with a strong experiential component and can be differentiated for teachers in teaching (activity-related) and subject (topic-related) enthusiasm (Kunter et al., 2011). For the higher education context, instructors’ enthusiasm (as perceived by students) has been found to be positively associated with teaching quality, student interest, and learning outcomes (Marsh, 1994; Kunter et al., 2008). In addition, findings for students also demonstrated that expressed positive emotions like enthusiasm are positively related to mastery goals and negatively related to performance avoidance goals (Huang, 2011).

Based on such results, we expect the mastery goals of university instructors to be positively associated with self-efficacy as well as enthusiasm and performance avoidance, and work avoidance goals to be negatively related to self-efficacy (cf. Wolters, 2003). For relational goals, we expect a positive association with self-efficacy and enthusiasm (Butler, 2012).

### Achievement Goals and Teaching Quality

In general, research on achievement goals backs up the notion that for diverse groups of people and domains (e.g., education, sport), overall achievement is positively related to mastery goals and mostly negatively related to performance avoidance goals (see Senko et al., 2011, for a detailed overview). In regard to school teachers, achievement goals have been shown to be associated with acquisition of teaching competences and instructional practices (Butler and Shibaz, 2008; Retelsdorf et al., 2010; Dresel et al., 2013), attitudes toward further training and professional learning (Butler, 2007; Runhaar et al., 2010; Nitsche et al., 2013b) as well as the experience of stress and work-load (Tönjes and Dickhäuser, 2009). For instance, Retelsdorf et al. (2010) showed that a teacher’s self-reported support of question asking and help seeking was positively related to mastery goals and negatively associated with performance approach goals. In addition, teachers with strong mastery goals reported using more instructional strategies that stimulate students cognitively and show them that the value of schoolwork lies in the acquisition of competences. Performance oriented teachers, however, reported using more instructional strategies that emphasize competition and achievement (Butler and Shibaz, 2008). These results are complemented by studies that assessed teaching quality using aggregated student ratings. Dresel et al. (2013) found that teachers with high mastery goals make more frequent use of cognitively and motivationally stimulating strategies of teaching. The student’s perspective constitutes a significant advantage, as the self-reports of teachers are often limited in their validity (Marsh, 2007).

Based on such results, we expected mastery goals of university instructors to be positively associated with teaching quality, and performance avoidance goals to be negatively associated with teaching quality.

### University Teaching and Assessments of Quality

When analyzing the associations of instructors’ achievement goals with teaching quality, it has to be considered that the latter is a complex construct that encompasses didactical as well as personal aspects of the instructor, and is conceived multidimensionally with a process and product dimension (Marsh, 1994). For the present context, the product dimension appears most relevant since it focuses on actual teaching outcomes in regard to the effect on students. It can be assessed by means of peer reviews or ratings through superiors or externals, though student evaluations of teaching (SETs) are most commonly used (cf. Marsh, 2007). Although they are widely accepted, student assessments of teaching quality can be distorted by an array of bias and unfairness variables (Greenwald, 1997), which often include aspects that cannot be directly influenced by the teacher, but do affect students’ perceptions—including prior knowledge, prior interest, perceptions of course relevance, year, age, and gender of the students, course size and format as well as age, gender, and career status of the instructor (see Marsh, 2007). It is well-established in the literature that SETs constitute a valid assessment of teaching quality when bias effects and the multi-level structure are reflected in the analysis model. If these constraints are controlled for, SETs provide a more accurate measure of teaching quality than instructor self-reports (Ting, 2000; Marsh, 2007).

### Research Questions

Building upon the theoretically and empirically determined usefulness of the concept of university instructors’ achievement goals, and to make this theoretical construct comprehensively accessible, the present study strived for an examination of the structure of goals (research question 1), the relationship of goals with other aspects of instructors’ motivations and personal characteristics (research question 2), and goal associations with teaching quality as perceived by students (research question 3).

#### Structure of Achievement Goals of University Instructors

In particular, we aimed to test whether instructors’ achievement goals are adequately represented by the five factors of mastery, performance approach, performance avoidance, work avoidance, and relational goals. Further, we expected instructors’ achievement goals to be moderately person-specific with a considerable course-specific proportion.

#### Relationships of Achievement Goals of University Instructors with Motivational and Biographical Characteristics

Secondly, we addressed the associations of achievement goals of university instructors with different motivational and biographical characteristics. Based upon the arguments given above, we expected achievement goals to be associated differently with age, gender, and career status. In addition, achievement goals were postulated to be differently related to other aspects of instructors’ motivations. **Table 1** provides an overview of the formulated hypotheses. Furthermore, we generally expect (the more functional) mastery goals to be stronger developed (i.e., higher means) than (the less functional) work avoidance goals.

**Table 1:** Overview of the hypotheses concerning the relationships of instructors’ achievement goals with other variables.

Achievement goals	Self-efficacy and enthusiasm	Age, gender, and career status	Teaching quality
Mastery	Positive associations with self-efficacy and enthusiasm	Lower for professors; higher for women	Positive association
Performance approach	(No directed hypotheses)	Higher for men	(No directed hypotheses)
Performance avoidance	Negative association with self-efficacy and enthusiasm	Higher for men	Negative association
Work avoidance	Negative association with self-efficacy and enthusiasm	Negative association with age; higher for men	Negative association
Relational	Positive association with self-efficacy and enthusiasm	Higher for women	Positive association


#### Associations of Achievement Goals of University Instructors with Teaching Quality

Our last research question addressed the relationship of achievement goals with distal student data. We expected instructors’ achievement goals to have a specific effect on teaching quality after controlling for other factors of instructors’ motivation and for bias variables potentially affecting SETs. In detail, we expected teaching quality to be higher when instructors had higher mastery, lower performance avoidance, lower work avoidance, or higher relational goals. Against the background of the heterogeneous prior findings for performance approach goals, we had no directed hypotheses regarding their relationship with teaching quality.

## Materials and Methods

### Procedure and Sample

University instructors were asked to answer a paper-and-pencil questionnaire assessing their achievement goals, self-efficacy, and enthusiasm with respect to a specific course. The respective students in these courses completed the student course evaluation questionnaires. Participation in multiple courses was possible for instructors and encouraged in order to yield first indications about the extent of course specificity of achievement goals. However, the study was not primarily designed to address this question—thus many instructors participated with a single course.

Altogether 251 university instructors (184 without Ph.D., 97 with Ph.D., thereof 51 full professors; 146 males, 92 females; mean age: 33.2 years) of a German university (from four departments: Philosophy and Social Sciences, Economic Sciences, Law, and Theology) participated in the study with a total of 392 courses among them (172 instructors participated with regard to one course, 42 with regard to two courses, and 37 with regard to three or more courses). Of these courses, 371 were also evaluated by students (total of 9,241 student assessments).

### Instructor Measurements

Internal consistencies for all instructor measurements were satisfying and can be found in **Table 2**.

**Table 2:** Descriptives and manifest correlations of instructors’ achievement goals, enthusiasm, and self-efficacy.

	(1)	(2)	(3)	(4)	(5)	(6)	(7)	(8)
Achievement goals								
(1) Mastery								
(2) Performance approach	0.13							
(3) Performance avoidance	0.13	0.82						
(4) Work avoidance	–0.11	0.24	0.22					
(5) Relational	0.39	0.16	0.17	–0.04				
(6) Self-efficacy	0.27	0.23	0.22	–0.16	0.31			
(7) Enthusiasm for teaching	0.27	0.06	0.09	–0.19	0.21	0.50		
(8) Enthusiasm for subject	0.24	0.08	0.06	–0.19	0.09	0.36	0.59	

Min	1.00	1.00	1.00	1.00	1.50	2.42	1.25	1.25
Max	8.00	8.00	8.00	8.00	8.00	8.00	8.00	8.00
*M*	6.34	5.62	6.09	2.96	5.90	6.27	7.06	6.70
*SD*	1.38	1.44	1.41	1.75	1.26	0.79	1.08	1.27
Skew	–1.03	–0.50	–0.94	0.72	–0.65	–0.85	–2.55	–1.70
Alpha	0.89	0.80	0.80	0.95	0.79	0.86	0.92	0.92


**N* = 230 instructors who responded with regard to a total of 392 courses. Coefficients were calculated on the course level. All |*r*| > 0.10 are significant at *p* < 0.05, |*r*| > 0.13 significant at *p* < 0.01.*

#### Achievement Goals

We adapted an established school teacher inventory by Butler (2012) and transferred it to the university context. Thereby, we specifically reformulated the items to fit the specifics of the university context. In order to assess achievement goals on a course-specific level, we asked the instructors to refer their answers exclusively to the current course and used the item stem “In this course…”. In line with Elliot (1999), we defined mastery goals as strivings to *develop own competences*^2^ (assessed with four items; sample item: “…I want to further develop my own competences”). For performance goals, we balanced their appearance and normative aspects within their approach and avoidance components by constructing two items for each combination. Based upon Urdan and Mestas (2006), approach-appearance goals were defined as striving to *appear competent to others* (two items; “…it is important to me to be perceived as competent”), and avoidance-appearance goals as wanting to *avoid looking incompetent* (two items; “…it is important to me to not be perceived as incompetent”). Following Grant and Dweck (2003), approach-normative goals were defined as *wanting to perform better than others* (two items; “…it is my goal to do well in comparison to fellow instructors”), and avoidance-normative goals as *wanting to avoid performing worse than others* (two items; “…it is my goal not to do badly in comparison to fellow instructors”). Symmetric wording was used when possible for approach and avoidance formulations (e.g., for the appearance components: “…to be perceived as competent”, “…to not be perceived as incompetent”). This conceptual decomposition allows for a balanced measurement of performance goals. For instance, performance approach goals were measured by using the mean of the four approach items, thus containing two items in regard to their appearance component and two items in regard to their normative component. Based on Butler (2012), work avoidance goals were defined as the striving *to cope with achievement situations with little effort* (four items; “…it is my goal to have to put in as little effort as possible”), and relational goals were defined as the wish *to create caring relationships with students* (four items; “…it is my main objective to establish a positive relationship with my students”). All items were answered on Likert type scales ranging from 1 (*do not agree at all*) to 8 (*agree completely*). They are available in the Supplementary Table.

#### Self-efficacy

An instructor’s self-efficacy beliefs toward teaching were assessed by adapting a scale from Nie et al. (2010) that asked how well instructors rated themselves in terms of instruction, motivation, and classroom management in the specific course of interest (e.g., for instruction, “How well can you provide an alternative explanation or an example when students are confused?”) on an 8-point Likert type scale (*absolutely not good—exceedingly good*).

#### Enthusiasm

By adapting scales from Kunter et al. (2011), we assessed instructors’ enthusiasm in regard to teaching (e.g., “I really enjoy working with the students in this course”) and in regard to subject (e.g., “I find this course’s scientific content interesting”) with four items each. They were answered on Likert type scales ranging from 1 (*do not agree at all*) to 8 (*agree completely*).

### Student Evaluation of Teaching Quality

The quality of teaching was assessed by using two subscales from the SEEQ (Marsh, 1982) which considered student (subjective) learning gains and overall assessment of teaching quality as central aspects of the teaching quality product. Student learning was captured with five items (e.g., “I have gained a lot of knowledge in this course”) and was answered on a Likert type scale ranging from 1 (*do not agree at all*) to 5 (*agree completely*) with an extra option *not assessable*. Student overall assessment of teaching quality consisted of four items (e.g., “How well does this instructor do in comparison to others?”) and was to be answered on a Likert type scale ranging from 1 (*poor*) to 5 (*very good*). Descriptive statistics reveal that both product dimensions of teaching quality, namely student learning (*M* = 3.79; *SD* = 0.75; α = 0.85; ICC2 = 0.90) and overall assessment of teaching quality (*M* = 3.94; *SD* = 0.82; α = 0.93; ICC2 = 0.92), were measured reliably.

As potential bias variables, a student’s year, gender, and prior interest (“Your level of interest in the subject prior to this course”) were assessed.

### Analyses

#### Confirmatory Factor Analyses

In order to test the structural hypothesis, Confirmatory Factor Analyses (CFA) were conducted with Mplus (Muthén and Muthén, 2012). Since the data violated the assumption of multivariate normality (Henze–Zirkler’s HZ = 1.090; *p* < 0.001; cf. Korkmaz et al., 2014), the MLR estimator was used (Yuan and Bentler, 2000). MLR adjusts the fit statistics based on Chi-Squared and the model standard errors—as such all estimates can be interpreted as usual. χ^2^ and SRMR were used as absolute fit indices, TLI as a relative fit index that also adjusts for parsimony, and RMSEA and CFI as non-centrality-based indices. To control for the construction design of the performance goal items, i.e., symmetrical formulation as well as balancing for appearance and normative components (which leads to shared method variance due to symmetric wording and item content), we *a priori* decided to model correlated errors between the corresponding items (Brown, 2015). Since, we had several instructors assessing more than one of their courses, we adjusted the standard errors by using the “type = complex” option in Mplus. We standardized the latent variables by setting their means to 0 and variances to 1.

#### Hierarchical Linear Models

A general methodological problem in regard to the analysis of students’ evaluations of university instructors is the determination of the appropriate level of analysis (see Cranton and Smith, 1990; Marsh, 2007). The data in course evaluations can be represented in a hierarchical structure compromising three levels: Student assessments (level 1) are nested within courses (level 2) conducted by instructors (level 3), who are—most of the time—responsible for multiple courses. Analyses conducted aggregated at the instructor level often result in a loss of information (e.g., within-instructor variance, within-course variance) and a diminishment of test power (Ting, 2000). In addition, students nested within courses are more homogeneous than observations from a non-nested study. This correlation of dependency violates the assumption of independence required for traditional statistics and can—already even when only mildly violated—lead to severe type I error biases (typically inflated; Kish, 1965). In order to avoid these problems, the associations between university instructors’ course-specific achievement goals with instructors’ biographical characteristics and student assessments should be analyzed by means of a multi-level approach that considerers all relevant levels simultaneously.

In order to test for achievement goal associations with biographical characteristics, we conducted two level regression analyses with age (treated as continuous variable), gender (male or female), and status (full professor: yes or no) as predictors on the instructor level (associations between these variables: *r* = –0.25 and –0.39) and instructors’ (course-specifically assessed) achievement goals as outcomes on the course level.

To examine the relationships of achievement goals with the two incorporated student assessments of teaching quality, we conducted two three level regression analyses. For each, we first analyzed the three-level null model for the dependent variables, which is defined through the three equations *Y*_ijk_ = π_0jk_ + *e*_ijk_(student level), π_0jk_ = β_00k_ + *r*_0jk_ (course level), and β_00k_ = γ_000_ + *u*_00k_(instructor level). This allows for a decomposition of the variance of the dependent variables into three levels, containing the variance within courses (*E*), the variance between courses (*R*_0_), and the variance between instructors (*U*_00_): *Var*(*Y*_ijk_) = *E* + *R*_0_ + *U*_00_ = *Var*(*e*_ijk_) + *Var*(*r*_0jk_) + *Var*(*u*_00k_). Based upon this, we controlled for distorting influences of potential bias and unfairness by expanding the null model for each of the two dependent variables. These models are defined through the equations *Y*_ijk_ = π_0jk_ + ∑_p_(π_pjk_ ⋅ *X*_pijk_) + *e*_ijk_ on the student level (*X*_p_ represents the *p*th predictor), π_0jk_ = β_00k_ + ∑_q_(β_0qk_ ⋅ *X*_qjk_) + *r*_0jk_ and π_pjk_ = β_p0k_ on the course level (*X_q_* represents the *q*th predictor), and β_00k_ = γ_000_ + ∑_s_ (γ_00s_ ⋅ *X*_sk_) + *u*_00k_ and β_0qk_ = γ_0q0_ on the instructor level (with *X*s as the *s*th predictor). Finally, instructors’ achievement goals as well as their self-efficacy and enthusiasm were inserted in the model.

We estimated all multi-level models using HLM 6 (Raudenbush et al., 2004) and restricted maximum likelihood estimation. Prior to the analyses, we *z*-standardized all continuous variables (dichotomous variables were coded as 0 and 1) and added all predictors grand-mean centered in the models (Enders and Tofighi, 2007). Consequently, the coefficients are fixed effects that can be interpreted similarly to standardized regression coefficients.

#### Missing Values

Missing values due to item non-response (less than 5.1% for all instructor items and less than 4.7% for all student items) were imputed using the expectation-maximization algorithm prior to all analyses (see Peugh and Enders, 2004).

## Results

### Preliminary Analyses

Inspection of descriptive statistics (cf. **Table 2**) revealed rather high means of the achievement goals (except for work avoidance goals), indicating that they constitute important aspects of the motivation of university instructors, and that university instructors frequently pursue multiple goals. Generally, with the exception of relational goals, the whole theoretically possible scale range was attained and quite large variances observed, indicating that there are substantial inter-individual differences in the personal importance of different goals. Comparing the within-instructor variances (i.e., the variances between courses of the same instructors) and the between-instructor variances (ICC) revealed preliminary indications that achievement goals are at least moderately person specific, but also depend to a substantial degree on the specific courses in which they are pursued. On a descriptive level, mastery (ICC = 0.61) and work avoidance goals (ICC = 0.74) were more person specific and performance and relational goals (ICC = 0.48–0.55) were more course specific.

Furthermore, a decomposition of the variance of both dimensions of teaching quality shows that 25–30% of the total variance can be attributed to differences between courses and that 44–46% of this between-course variance can again be ascribed to differences between instructors.

### Structure of Achievement Goals

**FIGURE 1:**
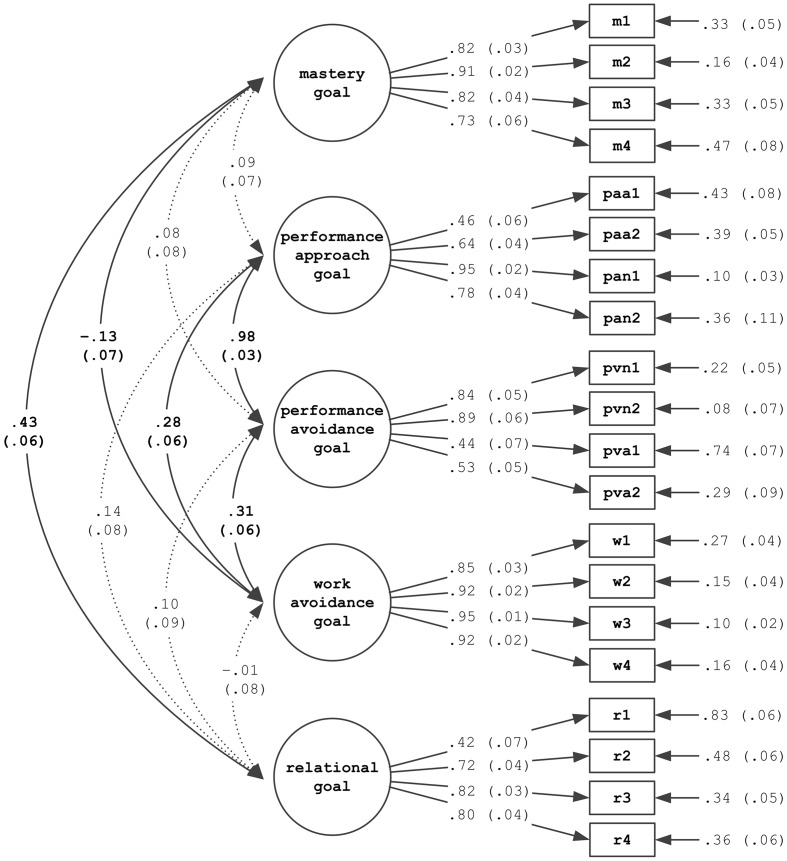
**Factor loadings and factor correlations of the five factor model (correlated errors are not displayed; non-significant factor correlations are displayed in dotted lines)**.

Estimation of the five factor model (differing between mastery, performance approach, performance avoidance, work avoidance, and relational goals) yielded a good fit to the data (χ^2^ = 358.56, *p* < 0.001, *df* = 156; CFI = 0.94; TLI = 0.93; RMSEA = 0.06; SRMR = 0.06). This indicates that these goal classes are principally distinguishable. **Figure 1** presents the factor loadings and latent correlations of this model. Remarkably, there was a very strong association between performance approach and performance avoidance goals. Comparing this five factor model to a four factor model with performance approach and avoidance goals collapsed (χ^2^ = 364.62, *p* < 0.001, *df* = 160; CFI = 0.93; TLI = 0.92; RMSEA = 0.06; SRMR = 0.07) showed that the differentiation between performance approach and avoidance had nevertheless a significantly better fit (evaluated using –2ΔLL rescaled difference in the model log-likelihood values: –2ΔLL(4) = 31.38, *p* < 0.001). On the basis of the five factor model, manifest means were calculated for the achievement goals used for the subsequent analyses.

### Relationship of Achievement Goals with Instructor Variables

The levels of achievement goals were different for different subgroups of our instructors (**Table 3**). Older instructors reported significantly lower work avoidance goals and higher relational goals than younger instructors, female instructors reported higher mastery as well as lower performance approach goals than males, and professors did not differ toward non-professors in their reported achievement goals.

**Table 3:** Relation of instructors’ achievement goals with age, gender, and career status.

	Instructors’ achievement goals				
	
Parameter	Mastery	Performance approach	Performance avoidance	Work avoidance	Relational
**Fixed effects**					
Intercept	-0.05 (0.06)	0.02 (0.06)	0.04 (0.06)	-0.04 (0.06)	-0.01 (0.06)
Level 2: Instructors					
Age	0.06 (0.07)	-0.08 (0.06)	0.01 (0.06)	-0.19^∗∗^ (0.06)	0.13^∗^ (0.07)
Gender (0 = male; 1 = female)	0.19^∗∗^ (0.06)	-0.11^∗^ (0.06)	-0.07^∗^ (0.06)	-0.08 (0.06)	0.06 (0.06)
Professor (0 = no; 1 = yes)	-0.08 (0.09)	-0.01 (0.07)	-0.07 (0.06)	0.05 (0.06)	-0.05 (0.07)
**Random parameters**					
Level 2					
Intercept (σ^2^)	0.40 (0.01)	0.45 (0.01)	0.51 (0.01)	0.28 (0.01)	0.49 (0.01)
Level 1					
Intercept (τ^2^)	0.62 (0.01)	0.50 (0.01)	0.42 (0.01)	0.66 (0.01)	0.49 (0.01)


**n*_*Courses*_ = 392, *n*_*Instructors*_ = 230. Presented are coefficients and their standard errors in parentheses. ^∗^*p* < 0.05; ^∗∗^*p* < 0.01.*

Concerning instructor self-efficacy, and enthusiasm for teaching and subject, there was a negative correlation with work avoidance goals and a positive correlation with mastery goals and relational goals (not significant for enthusiasm for the taught subject). Performance approach and performance avoidance goals were positively related to self-efficacy, but not to enthusiasm.

### Associations of Achievement Goals with Teaching Quality

Presented in **Table 4** are the results of estimating the three level models, including instructors’ achievement goals, and self-efficacy and enthusiasm as predictors while concurrently controlling for student, course, and instructor bias variables. Mastery goals constituted a positive predictor for student learning and the overall assessment of teaching quality whilst performance avoidance goals were a negative predictor for both of these product dimensions of teaching quality. Also, work avoidance goals were a negative predictor for student learning (however, only marginally significant for the overall assessment of teaching quality).

**Table 4:** Relations of instructors’ achievement goals, self-efficacy, and enthusiasm with student evaluations of teaching quality.

	Students’ ratings of teaching quality	
	
Parameter	Student learning	Overall assessment
**Fixed effects**		
Intercept	0.29 (0.05)	0.21ˆ* (0.06)
Level 1: Students		
Gender (0 = male, 1 = female)	-0.06ˆ*** (0.02)	-0.05ˆ* (0.02)
Year	-0.01 (0.01)	-0.01 (0.02)
Previous Interest	0.26ˆ*** (0.01)	0.16ˆ*** (0.01)
Level 2: Courses		
Achievement goals		
Mastery	0.05ˆ* (0.03)	0.06ˆ* (0.03)
Performance approach	0.01 (0.04)	0.02 (0.05)
Performance avoidance	-0.07ˆ* (0.04)	-0.09ˆ* (0.05)
Work avoidance	-0.06ˆ* (0.03)	-0.04ˆ+ (0.03)
Relational	0.01 (0.02)	0.05 (0.04)
Self-efficacy	0.10ˆ** (0.03)	0.08ˆ** (0.04)
Enthusiasm for teaching	0.01 (0.04)	0.08ˆ* (0.05)
Enthusiasm for subject	0.07ˆ* (0.03)	0.01 (0.03)
Course size	-0.01ˆ* (0.01)	-0.01 (0.01)
Department 1 (0 = no, 1 = yes)	-0.05 (0.11)	-0.16 (0.14)
Department 2 (0 = no, 1 = yes)	-0.17ˆ* (0.08)	-0.19ˆ* (0.09)
Department 3 (0 = no, 1 = yes)	0.07 (0.07)	0.06 (0.09)
Level 3: Instructors		
Age	0.02 (0.03)	-0.01 (0.04)
Gender (0 = male, 1 = female)	-0.02 (0.03)	-0.11ˆ** (0.04)
Professorship (0 = no, 1 = yes)	-0.03 (0.03)	-0.07ˆ* (0.04)
**Random parameters**		
Level 3		
Intercept (σ^2^)	0.68 (0.01)	0.67 (0.01)
Level 2		
Intercept ( 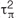 )	0.09 (0.01)	0.10 (0.02)
Level 1		
Intercept ( 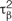 )	0.09 (0.02)	0.14 (0.03)


**n*_*Students*_ = 9,241, *n*_*Courses*_ = 371, *n*_*Instructors*_ = 230. Unless otherwise noted, the predictors are *z*-standardized based on the grand mean. The four different departments were dummy coded with three binary variables. Presented are coefficients and their standard errors in parentheses. Since we had directed hypotheses, the significance tests of all variables, except performance approach goals, were one-tailed (Snijders and Bosker, 1999). ^+^*p* < 0.10; ^∗^*p* < 0.05; ^∗∗^*p* < 0.01; and ^∗∗∗^*p* < 0.001.*

Apart from these findings that are central to the research questions at hand, we found that self-efficacy significantly predicted both dimensions of teaching quality. Also, enthusiasm for teaching constituted a predictor for the overall assessment of teaching quality but not for student learning while enthusiasm for subject predicted student learning but not the overall assessment of teaching quality.

### Exploratory Analyses of Performance Appearance and Performance Normative Goals

Due to the particularly large correlation between performance approach and performance avoidance goals, and the delineated theoretical relevance of the appearance and normative components of performance goals, we analyzed *post hoc* the latter distinction under an exploratory focus. Thus, we estimated an alternative five factor model including the appearance component and the normative component of performance goals as separate factors—instead of distinguishing between performance approach and performance avoidance goals. The fit of this alternative model was just as good as that of the original model (χ^2^ = 334.17, *p* < 0.001, *df* = 156; CFI = 0.95; TLI = 0.93; RMSEA = 0.06; SRMR = 0.06), whilst the latent correlation dropped from γ = 0.98 (between approach and avoidance goals) to γ = 0.72 (between appearance and normative components). Differentiating between appearance and normative components of performance goals also revealed differential relationships with other variables that are theoretically sensible (no differential relationships were evident for performance approach and performance avoidance goals). Performance appearance goals were positively associated with mastery goals (γ = 0.25; *p* = 0.002) and relational goals (γ = 0.26; *p* = 0.001), while performance normative goals were not (γ = 0.07–0.17; *p* > 0.22). Moreover, performance normative goals were higher for men than for women (*b* = –0.11; *SE* = 0.06; *p* = 0.03), while performance appearance goals were not (*b* = –0.04; *SE* = 0.06; *p* = 0.48). Both goals did not differ depending on instructor age (|*b*| < 0.11; *SE* = 0.06; *p* > 0.07) or status (|*b*| < 0.09; *SE* = 0.07; *p* > 0.17). Finally, performance normative goals posed a negative predictor toward the overall assessment of teaching quality (*c* = –0.09; *SE* = 0.04; *p* = 0.02) and, in tendency, toward students’ learning (*c* = –0.06; *SE* = 0.04; *p* = 0.08). In contrast to that, for performance appearance goals, no relationships with students’ evaluations of teaching quality were evident (*c* > –0.05; *SE* = 0.04; *p* > 0.21).

## Discussion

The present research addressed university instructors’ achievement goals. Specifically, we investigated the structure of achievement goals and their relationship with other instructor variables and teaching quality. Strengths of the present work include its innovative focus on university instructors’ achievement goals, the consideration of multiple outcomes, and the incorporation of both instructor as well as student data. In general, the results support the idea that the achievement goals approach is suitable to describe and explain important driving motivational forces underlying instructors’ cognition and behavior in teaching.

The preliminary evidence of the present study regarding course specificity indicated that achievement goals were moderately person specific while at the same time showing differential fluctuations across courses. This complements the sparse results for the specificity of pupils’, students’, and school teachers’ achievement goals which indicate that achievement goals have domain-unspecific parts—which can be linked to individual personality—as well as domain-specific parts that are more influenced by contextual conditions (Bong, 2001; Fryer and Elliot, 2007; Sparfeldt et al., 2007; Fasching et al., 2011). Of course, these results are limited in that not all university instructors in our sample taught and made assessments in regard to more than one course. Thus, it is uncertain in how far this led to a biased sample for the analyses. Conducting a study that is specifically designed to address the specificity of instructors’ achievement goals is a possible perspective for future research. Despite this limitation, the results can be taken as a notable hint for the course specificity of the teaching motivation of university instructors.

Concerning the structure of the achievement goals of university instructors (research question 1), it is established that principally, mastery, performance approach, performance avoidance, work avoidance, and relational goal classes can be verified as being definable separately from each other (as proposed by Butler, 2012, for school teachers). This indicates that these goal classes are principally distinguishable. In addition, performance approach and performance avoidance goals were differentially related to teaching quality. However, the factor correlation between performance approach and avoidance goals was very high. This pattern may be interpreted as resulting from the specific characteristics of the achievement situation in which instructors (and teachers) are embedded. Instruction by university instructors (and teaching of school teachers) nearly always and continuously takes place directly in the public of the course (or the classroom, respectively). Thus, demonstrating good performances and avoiding bad performances may be two sides of the same coin for them, and the only way to avoid being considered a bad instructor may be to appear as a good teacher (opposed to students in achievement situations which may, for example, continuously fear having bad performances, but may decide only from time to time to show what they know). This might explain the high correlations for university instructors and school teachers, and be a first hint that the classic approach–avoidance distinction may be less important in the instruction context than the appearance–normative distinction. This circumstance should be explicitly investigated in future research.

Relations with other predicted personal variables could generally be confirmed (research question 2). Concerning the expected associations with biographical characteristics (age, gender, and career status), several of our hypotheses were confirmed: Older instructors pursued fewer work avoidance goals and males reported lower mastery but higher performance goals as compared to women. However, we did not find gender differences toward the pursuing of relational goals. This initially comes as a surprise, since women frequently put a stronger focus on interpersonal encounters (Acker and Feuerverger, 1996; Butler, 2012, 2014). However, it can be assumed that due to the low density of interaction (usually one session per week), women seek to pursue their assumed higher wish for meaningful interpersonal relationships not primarily through their courses but through other means (e.g., interactions with colleagues, private interactions). In addition, we did not find that women and men differ in their pursuit of work avoidance goals. Also, professors did not differ from non-professors in their achievement goals. This indicates that for future research, it might be more interesting to include more concrete group aspects (such as subject or teaching load) and to investigate possible interactions between them (e.g., gender differences might vary from subject to subject). Concerning instructors’ self-efficacy and enthusiasm, we found the expected positive correlations with mastery and the expected negative correlations with work avoidance goals. For relational goals, we only found a positive correlation for teaching related enthusiasm, but not for subject related enthusiasm. However, since relational goals focus on the addressees and thus on the teaching process itself but not the subject (Butler, 2012), this appears sensible. Both performance approach and performance avoidance goals were positively related to self-efficacy, yet not to enthusiasm. This rejects our hypotheses toward the effect of performance avoidance goals (which we expected to be negatively associated with functional aspects of instructor motivation). However, due to the high correlation between performance approach and performance avoidance goals, a unique effect of performance avoidance goals may be masked (cf. Linnenbrink-Garcia et al., 2012). All in all, the results underline that different groups of university instructors have differently formed motivational systems toward teaching.

To examine the relationships of achievement goals with instructors’ teaching quality, we gathered students’ assessments regarding knowledge gains and overall assessment of teaching quality (research question 3). The results confirmed the expected positive associations of mastery goals with both of the two product dimensions of teaching quality included in the present study. It might be reasonably assumed that following the insights of research regarding school teachers, university instructors, who want to learn something themselves (i.e., having strong mastery goals), use to a greater extent teaching strategies that cognitively stimulate students and support student acquisition of competencies (Meece, 1991; Retelsdorf et al., 2010). This in turn can be assumed to directly influence student learning and their overall assessment of teaching quality. In contrast, performance avoidance goals were negatively related to both teaching quality dimensions. This is in line with the results of research on school teachers, indicating that performance avoidance goals are associated with the use of less adequate instructional strategies (i.e., strategies that stress competition and achievement and not so much the development of student competence; Butler and Shibaz, 2008; Dresel et al., 2013). In the university context, it can further be assumed that in order to realize performance avoidance goals, it is helpful for instructors to employ teacher-centered instruction instead of student-centered instruction. This is because student-centered instruction might make oneself more vulnerable to being exposed to (unexpected) topics or situations one does not know much about. This in turn might cause lower student outcomes. Apart from this, work avoidance goals were negatively related to student learning. This corresponds to findings showing that higher work avoidance goals are accompanied by less effort (Urdan, 1997) as well as less interest in teaching and contempt of help and support (Retelsdorf et al., 2010; Nitsche et al., 2011), factors which can ultimately result in lower student learning. The hypothesis on the association of relational goals with the overall assessment of teaching quality could not be confirmed. This might be due to the consideration of bias variables having already controlled for the expected rating bias (i.e., students rated instructors’ teaching quality differently depending on how much they like them)—which was assumed to be especially strong for relational goals since they directly influence how much students like their instructor and, in turn, how well they rate them (Marsh, 2007). Overall, the associations between instructors’ achievement goals and student evaluations of their teaching quality turned out to be rather small. However, they are in the range known from research on the associations between school teachers’ goals and student perceptions of their classroom instruction (e.g., Butler and Shibaz, 2008; Dresel et al., 2013), and research on the associations between university instructor characteristics and student evaluations of teaching quality (cf. Marsh, 2007). Thus, the associations of university instructors achievement goals identified in the present study can be seen as indicative for relevant motivational influences on teaching quality in higher educational contexts.

Toward the other aspects of instructor motivation, the results asserted that considering one’s teaching skills as high (i.e., having a strong self-efficacy) was associated with high student learning and overall assessment (Morris and Usher, 2011). Also, results showed that enthusiasm had a distinct relationship with the dependent variables: Whilst learning was only predicted by enthusiasm for subject, the overall assessment was only predicted by enthusiasm for teaching. The results are in line with the dimensionality presented by Kunter et al. (2011) and indicate that having an instructor who is enthusiastic about his or her subject but not about teaching results in higher student learning, though this is not reflected in the overall assessments made by students regarding teaching quality.

Although, literature on school teachers typically reports high correlations between performance approach and performance avoidance goals as well (e.g., γ = 0.88; Nitsche et al., 2011), their association was so high in the present study that it must be seen as questionable to justify their differentiation in these two factors. An additional indication against the distinction in solely approach-avoidance for university instructors’ performance goals is the positive correlation between performance avoidance goals and teaching self-efficacy (which stays in sharp contrast to the negative correlations found for students; cf. Elliot, 2005). The therefore conducted additional exploratory analyses advised consideration of instructors’ performance goals in a more differentiating manner. Results indicated that the consideration of appearance and normative components might be at least as important for the population of instructors as the (well-established) differentiation in approach and avoidance dimensions (cf. research question 1). Additionally, the splitting of performance goals in appearance and normative components resulted in a more theory compliant correlation pattern including more specific relationships for the appearance and normative components of performance goals than for the approach and avoidance performance goals of instructors. Also (cf. research question 2), with its distinct effect patterns toward enthusiasm, the differentiation between appearance and normative goals proved potent and theoretically sensible, since being concerned with how well one does in comparison to others need not be related to one’s enthusiasm. Males reported higher performance normative goals than women, indicating that they strive more to perform well in comparison with others. This complies with results on gender disparities in the university context (Acker and Feuerverger, 1996). Toward their relation to the two product dimensions of teaching quality (cf. research question 3), no differential hypotheses were formulated regarding the distinction of performance goals in appearance and normative component for the explanatory analyses. However, the results indicated that normative goals were a significant, negative predictor of the overall assessment of teaching quality, implying that instructors, who are worried about how well they do in comparison to other instructors, are actually worse at instruction. This might be traced back to antecedents of the respective goal (e.g., own competences being perceived as low might lead to concerns about normative performance in the first place), a less favorable focus when teaching (i.e., not the students but other instructors are focused on), or, concomitantly, a higher degree of avoidance motivation, which is usually associated with worse performance (Elliot, 1999).

Taken together, the present study provides strong evidence toward the structure of achievement goals and their relationship with other instructor variables and teaching quality, especially by including student data and a multivariate structure. The results endorse that achievement goals are suitable to describe and explain important driving forces underlying an instructor’s cognition and behavior. Nevertheless, the results also indicate that more research especially in regard to performance goals is required. Both differentiations of performance goals (approach–avoidance; appearance–normative) described the data only slightly better than one factor. Nevertheless, we found differential associations with other variables indicating unique variances of these components (cf. Linnenbrink-Garcia et al., 2012). In light of the present results, it therefore seems reasonable to assume that systematically combining approach and avoidance dimensions with appearance and normative components advances the clarification of their characteristics and functioning. This might help resolving the ongoing debate about the effects of performance goals (Senko et al., 2011).

Besides the already mentioned aspects, some limitations of the present work should be kept in mind when interpreting their results. The sample was recruited from only one university (though from a variety of departments), the cross-sectional design only allows for statements about coherences but not causal inferences (although specific directions, e.g., an effect of instructors’ goals on students’ perceptions of teaching quality seem theoretically more reasonable than the reverse direction), and several measures might be affected by social desirability and should therefore be interpreted carefully (e.g., as seen in the low means of work avoidance goals).

Although more work is required to thoroughly understand the achievement goals of university instructors, some preliminary practical implications can already be delineated: For instance, professional development of university instructors should focus on developing and enhancing mastery goals (cf. Urdan and Turner, 2005), while at the same time it might be beneficial to support instructors in dealing with high work load by other means instead of pursuing work avoidance goals (e.g., prioritizing, using different resources). Since the relationships of performance approach and performance avoidance goals are theoretically and empirically still unclear, practical implications toward performance goals can not yet be drawn.

## Conclusion

Despite limitations, the present results allow us to conclude that university instructors pursue a bundle of distinct achievement goals that vary inter-individually and intra-individually in strength, and differentially predict cognition and behavior in university lecturing. The results largely confirm the postulated structure and point toward the relevance of individual dimensions of university instructors’ achievement goals. This emphasizes the importance of the theoretical concept of instructors’ achievement goals for the analysis of instruction and learning in higher education. Consequently, the study at hand encourages future research on university instructors’ achievement goals.

## Author Contributions

All authors listed, have made substantial, direct, and intellectual contribution to the work, and approved it for publication.

## Conflict of Interest Statement

The authors declare that the research was conducted in the absence of any commercial or financial relationships that could be construed as a potential conflict of interest.

## Footnotes

1 In addition to these five dimensions, mastery avoidance goals (strivings to avoid incomplete knowledge or intrapersonal incompetence) have also been suggested, but are a relatively new construct that is not yet established in the achievement goal literature on teachers (Elliot, 1999; Pintrich, 2000a).

2 As such, they focused on the improvement of competences and did not include—the also often operationalized—items related to curiosity (e.g., Lepper et al., 2005), interest (e.g., Midgley et al., 1998), or task (e.g., Barron and Harackiewicz, 2001).
