# Supplementary material for: Structure and Relationships of University Instructors’ Achievement Goals
Source: Front Psychol. 2016 Mar 23;7:375. doi: 10.3389/fpsyg.2016.00375 (PMC4803730; doi:10.3389/fpsyg.2016.00375)
Supplement: Supplementary file 1 [file Table_1.PDF]

Supplementary Material

Structure and Relationships of University Instructors' Achievement Goals

Martin Daumiller*, Robert Grassinger, Oliver Dickhäuser, Markus Dresel

*** Correspondence:** Corresponding Author: Martin.Daumiller@phil.uni-augsburg.de

1 Supplementary Table

Items to Assess Instructors' Achievement Goals (original German items in squared brackets)

Item stem: In this course [In dieser Lehrveranstaltung],…
Mastery
it is important to me to acquire further knowledge and competences [ist es mir wichtig, etwas Neues dazuzulernen].
it is one of my objectives to also learn new things myself [geht es mir darum, auch selbst etwas dazuzulernen].
it is my goal to enhance my factual and didactic knowledge [ist es mein Ziel, mein fachliches und didaktisches Wissen zu erweitern].
I want to further develop my own competences [will ich meine eigenen Kompetenzen weiterentwickeln].
Approach-appearance
it is important to me to be perceived as competent [ist es mein Ziel, als kompetent wahrgenommen zu werden].
it is one of my objectives to be perceived by students as a good instructor [möchte ich, dass die Studierenden merken, wie gut ich als Dozent(in) bin].
Approach-normative
it is my goal to do well in comparison to fellow instructors [möchte ich im Vergleich zu anderen Dozent(inn)en gut abschneiden].
I want my teaching to be rated better than fellow instructors' [will ich, dass meine Lehre besser bewertet wird als die Lehre von Kolleg(inn)en].
Avoidance-appearance

it is important to me to not be perceived as incompetent [ist es mir wichtig, nicht als inkompetent wahrgenommen zu werden].

it is one of my objectives not to teach in a way that is perceived badly by the students [ist es mir vor allem wichtig, keine Lehre zu machen, die bei den Studierenden schlecht ankommt].

Avoidance-normative

it is my goal not to do badly in comparison to fellow instructors [möchte ich im Vergleich zu anderen Dozent(inn)en nicht schlecht abschneiden].

I don’t want to have worse teaching quality ratings than fellow instructors [will ich, dass meine Lehre nicht schlechter als die Lehre von anderen Dozent(inn)en bewertet wird].

Work avoidance

it is important to me to have the least possible preparation time [ist es mir wichtig, eine möglichst geringe Vorbereitungszeit zu haben].

it is one of my objectives to practice my teaching with the least possible effort [möchte ich Lehre mit möglichst wenig Aufwand betreiben].

it is my goal to have to put in as little effort as possible [ist es mein Ziel, einen möglichst geringen Arbeitsaufwand zu haben].

I want to have to do as little work as possible [will ich möglichst wenig tun müssen].

Relational

it is important to me to achieve a personal connection with students [ist es mir wichtig, mit den Studierenden auch auf eine persönliche Ebene zu kommen].

it is one of my objectives to establish a partner-like relationship with students [ist es eines meiner wichtigsten Ziele, ein partnerschaftliches Verhältnis mit den Studierenden aufzubauen].

I want to signal to my students that I have a genuine interest in their opinions and perceptions [ist es mein Ziel, den Studierenden zu signalisieren, dass ich echtes Interesse an ihren Meinungen und Auffassungen habe].

it is my main objective to establish a positive relationship with my students [strebe ich vor allem danach, ein gutes Verhältnis mit den Studierenden zu entwickeln].

Note. Item texts are a translation of the original German items and are not validated in the English-speaking context.
